# Going Up or Sideways? Perception of Space and Obstacles Negotiating by Cuttlefish

**DOI:** 10.3389/fphys.2017.00173

**Published:** 2017-03-27

**Authors:** Gabriella Scatà, Anne-Sophie Darmaillacq, Ludovic Dickel, Steve McCusker, Nadav Shashar

**Affiliations:** ^1^Eliat Campus, Department of Life Sciences, Ben-Gurion University of the NegevBeer-Sheva, Israel; ^2^Queensland Brain Institute, University of QueenslandSt. Lucia, QLD, Australia; ^3^EThOS Ethologie Animale et Humaine (UMR, Centre National De La Recherche Scientifique 6552), Team Cognitive Neuro Ethology of Cephalopods, Normandie UniversitéCaen, France

**Keywords:** space perception, cuttlefish, cephalopod, obstacles negotiation, three-dimensional space

## Abstract

While octopuses are mostly benthic animals, and squid prefer the open waters, cuttlefish present a special intermediate stage. Although their body structure resembles that of a squid, in many cases their behavior is mostly benthic. To test cuttlefish's preference in the use of space, we trained juvenile *Sepia gibba* and *Sepia officinalis* cuttlefish to reach a shelter at the opposite side of a tank. Afterwards, rock barriers were placed between the starting point and the shelter. In one experiment, direct paths were available both through the sand and over the rocks. In a second experiment the direct path was blocked by small rocks requiring a short detour to by-pass. In the third experiment instead, the only direct path available was over the rocks; or else to reach the goal via an exclusively horizontal path a longer detour would have to be selected. We showed that cuttlefish prefer to move horizontally when a direct route or a short detour path is available close to the ground; however when faced with significant obstacles they can and would preferentially choose a more direct path requiring a vertical movement over a longer exclusively horizontal path. Therefore, cuttlefish appear to be predominantly benthic dwellers that prefer to stay near the bottom. Nonetheless, they do view and utilize the vertical space in their daily movements where it plays a role in night foraging, obstacles negotiation and movement in their home-range.

## Introduction

Navigation has been extensively studied in two-dimensional environments, where the animal has to locate a goal by moving across a horizontal surface, neglecting the vertical dimension. However, the world is three-dimensional and since all animals have to move along the vertical plane at some point, they need to take the vertical component of space into account. The importance of vertical space has indeed recently been recognized for the conservation of several species, as well as for the welfare of animals kept in captivity (O'Neill-Wagner, 1994; Clarence et al., 2006; Tracey et al., 2014). Taking the vertical dimension into account makes navigation more complex; for example, the amount of space to be represented is larger than when encoding a planar two dimensional environment (Jeffery et al., 2013). This is especially true for animals that are able to move from and to any point in a volumetric space.

It has been suggested that the locomotor style of an animal is correlated with the accuracy with which spatial information in the horizontal and vertical planes is encoded and which of this information is prioritized (Flores-Abreu et al., 2014). Animals able to move freely in the three dimensions (e.g., fish, bats, bees, birds) encode the vertical information with either equal or higher accuracy than the horizontal information and seem to prefer vertical to horizontal information, while animals constrained to a surface (e.g., rats) do the opposite (Hurly et al., 2010; Holbrook and Burt de Perera, 2013; Davis et al., 2014; Flores-Abreu et al., 2014; but see Ulanovsky, 2011; Savelli and Knierim, 2013; Yartsev and Ulanovsky, 2013; Scatà et al., 2016). However, species of bees that differ in their use of vertical space also differ in the accuracy with which they learn height and in their ability to communicate this information (Nieh et al., 2003; Dacke and Srinivasan, 2007; Eckles et al., 2012). In addition, the performance of a number of species of birds in solving a detour task exclusively on the ground seems to be correlated with the extent to which they move vertically. Canaries for example, which are more used to move in all three-dimensions and fly over barriers, find it almost impossible to detour around a ground obstacle (Zucca et al., 2005). This suggests that the way animals negotiate obstacles reflects the degree to which they normally exploit the vertical vs. horizontal space. Thus, it could be the ecology of the species and its main plane of behavior, or as Nardi and Bingman suggest “its 3D occupancy profile” (Nardi and Bingman, 2013), that explains which component of spatial information is the most relevant to and preferred by the animal.

In previous experiments, we showed that *Sepia officinalis* cuttlefish, which are mostly benthic but can also move freely in a volumetric space, are able to learn spatial information in the vertical dimension, and prefer vertical over horizontal spatial cues when faced with conflicting situations (Scatà et al., 2016). Similar results have been reported for both benthic and pelagic fish (Holbrook and Burt de Perera, 2011; Davis et al., 2014). This dominance of vertical spatial information in fish was suggested to depend on the ability of fish to detect changes in hydrostatic pressure, a salient cue unique to vertical space (Davis et al., 2014; Holbrook and Burt de Perera, 2011). However, cuttlefish buoyancy system is mostly independent of depth (Webber et al., 2000) and pressure sensitivity in other cephalopods appears to be quite low (Rice, 1964; Jordan, 1988). Alternatively, it is possible that cuttlefish are more likely to use vertical information because their main activities - vigilance from predators, foraging, and movement—are performed along the vertical plane (Barbosa et al., 2008; Ulmer et al., 2013).

Cephalopods present a full range of use of space. While most octopus species are mostly benthic (though there are fully pelagic species), and squids are neritic to pelagic, cuttlefish present an in-between case where they spend most of their time as benthic predators, yet move up into the water column at will (Hanlon and Messenger, 1996). Indeed, although known as bottom-dwellers, cuttlefish were reported to become neutrally buoyant and move upwards in the water column at night (Denton and Gilpen-Brown, 1961; Wearmouth et al., 2013). This diel migration pattern has been observed mainly in laboratory conditions, with Aitken et al. (2005) reporting it in the field by tracking the giant Australian cuttlefish (*S. apama*), which moves deeper at night. Little is known about the navigational strategies of cuttlefish and whether or not they move equally in all three dimensions, or have a preferred dimension of locomotion. Their movement patterns have rarely been investigated, especially along the vertical plane. When presented with a vertical wall maze with only two escape holes, one lower and to the left and the other higher and to the right (10 and 60 cm from the bottom, respectively), cuttlefish escaped mostly by the lowest hole (13 out of 18 cuttlefish) (Karson et al., 2003). This setup required cuttlefish to swim upwards at least 6 body heights to escape through the top hole. Thus, it is interesting that a small percentage of the tested animals (5 out of 18) still selected the top hole and maintained such preference across trials.

In the current study, we examined the use of three-dimensional space in the cuttlefish *Sepia officinalis* and *S. gibba* in daytime spatial orientation. In particular, we investigated the use of horizontal versus vertical paths in a navigational task in which the animal has to negotiate a barrier to reach a shelter. We also examined the use of vertical space as time spent at different water depths at night by *S. gibba* cuttlefish, for which data on this is absent in the literature. *S. officinalis* is a nekton-benthic species which is mostly found on sandy or rocky bottoms from shallow coastal water (2–3 m depth) to 200 m depth (Guerra, 2006). *S. gibba* is associated with coral reefs, a more complex three-dimensional environment, which also requires more agility and maneuvering skills (Jastrebsky et al., 2016). It can be found in very shallow waters (1 m), yet not much is known of this species (Reid, 2005). A difference in the use of vertical routes between these two species could relate to the degree of vertical complexity of their natural habitat.

## Materials and methods

Two different setups were used for testing young *Sepia officinalis* and *S. gibba* cuttlefish, each examining a different level of complexity in obstacles negotiation. *S. officinalis* were raised and tested in France while *S. gibba* were examined in Israel. Therefore, each experimental setup is presented separately. The movement and use of vertical space of *S. gibba* cuttlefish only was analyzed at night.

### *S. officinalis* experiments

#### Subjects

Sixteen young [7–8 weeks old, mantle length of 15–20 mm, about 5 mm tall (body height, bh) and 8 mm wide] *S. officinalis* cuttlefish took part in an experiment testing their path preference while bypassing barriers. Cuttlefish were hatched from eggs collected in the vicinity of Luc-sur-Mer (France). Eggs, initially laid in clusters, were separated to ensure optimal development and were put in shallow tanks at the Centre de Recherches en Environnement Côtier (CREC, Luc-sur-Mer, France). All tanks were supplied with running oxygenated sea water at 17 ± 1°C. After hatching, cuttlefish were first housed in small groups and then, 1 week before experiments began, housed in individual tanks. They were provided with enriched habitats following previous studies which showed that an enriched environment facilitates development of learning and memory capabilities in young cuttlefish (Dickel et al., 2000; Poirier et al., 2004, 2005). These enriched habitats consisted of tanks with rocks, plastic seaweed and PVC tube as shelters. Animals were fed daily with live shrimp (*Crangon crangon*) and crabs (*Carcinus maenas*) of suitable size.

#### Experimental setup—experiment 1

**Figure 1:**
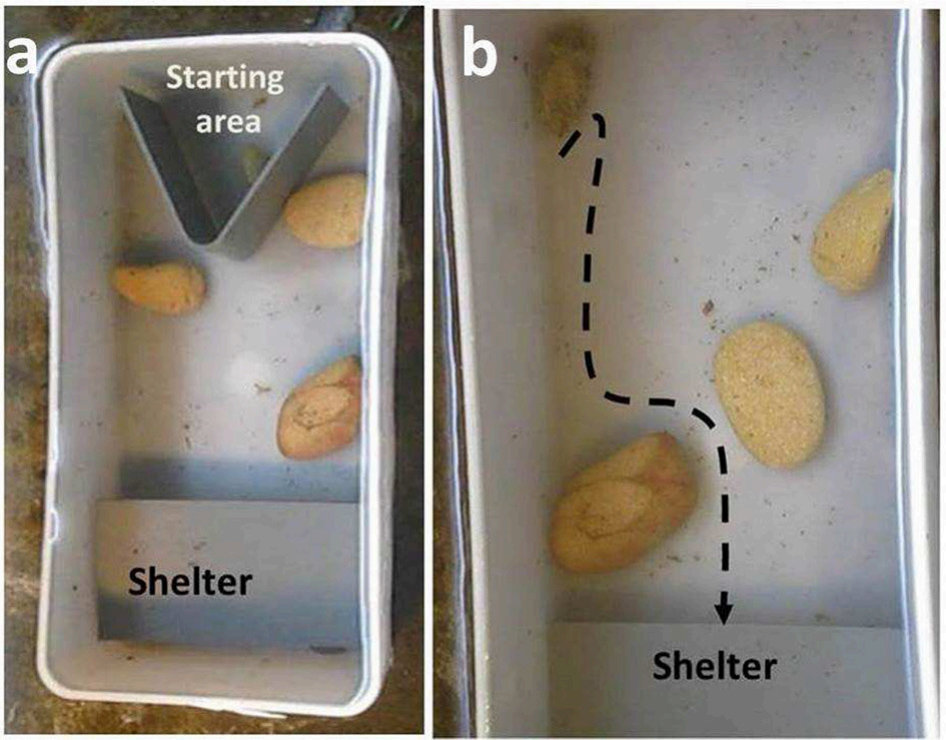
**Experiment 1- Rock obstacles: (a)** training setup, **(b)** testing setup. Cuttlefish were set at the starting area and after settling on the bottom of 30 sec. were allowed to move into the shaded shelter. In training sessions the rocks were set along the sides of the tank and a direct line to the shelter was available. In the testing setup the rocks prevented such a direct line and the animals had to go around or above them. Dashed line shows the path of the cuttlefish in this image.

Training and experiments took place in the same tank (Figure 1). Tank was made of opaque white plastic 20 × 10.5 × 7 cm (length × width × height). The tank was filled with seawater to its top. A 5 cm wide shelter was set at one side of the tank, with the dimensions of 5 × 10.5 cm. 3 low (no more than 1.5 cm in height) but wide stones were positioned in the tank. In the training session the stones were set along the sides of the tank, 2 in one side and one on the other. During the test, the stones were set diagonally in the tank such that they blocked any direct path, but could be negotiated and passed by going around them. Direction of the diagonal was changed randomly.

Training and testing took place in the same water table in which the animals were raised. Hence the animals experienced the same lighting and temperature conditions. Seawater was replaced between each training/experimental run to prevent possible odor cues.

#### Training and testing—experiment 1

Cuttlefish were given 3 training presentations to learn to reach the shelter at the opposite side of the experimental tank, with the training setup of the stones obstacles (Figure 1a). In each run, the animal was placed in the “starting area” at one side of the tank. Once the animal had settled and after at least 30 s, the wall was raised, and the animal was allowed to move to the shelter. After the animal had reached the shelter, it was rewarded with a 5-min rest in it, after which it was returned to the holding tank.

After having 3 training runs the animal was tested once with a different configuration of the obstacle stones (Experiment 1) (Figure 1b). This time there was no direct line to the shelter but the animal had to choose how to path them. It could stay on the bottom and go around the stones or it could go up and swim over them.

Each animal had no more than 2 sessions per day with at least 4 h between them. Training and testing sessions were videotaped from above.

### *S. gibba* experiments

#### Subjects

Twelve juvenile *S. gibba* were used in two experiments (Experiment 2 and Experiment 3). Four of the *S. gibba* cuttlefish used for experiment 2 and experiment 3 were used for the night observation experiment. The animals were reared from wild-caught eggs up to 2 months old. During rearing cuttlefish were housed as a group, in an indoor holding tank (40 × 36 × 20 cm; width × length × height, 18 cm water level) with running seawater at sea temperature, at the Underwater Observatory in Eilat, Israel. At 2 months of age, when the animals were about 15 mm mantel length and about 5 mm tall (body height) and 8–9 mm wide, they were transferred into a different holding tank of running seawater in the outdoor facilities of the Inter-University-Institute of Eilat (IUI). Both tanks had a sandy bottom, shelters, and rocks to provide an environment resembling natural conditions as much as possible. This enriched environment promotes learning in cuttlefish (Dickel et al., 2000; Poirier et al., 2004, 2005). Animals were fed with shrimps (*Artemia*), which were administered such that food was constantly available in the tank (*ad libitum*).

#### Experimental setup—experiments 2 and 3

**Figure 2:**
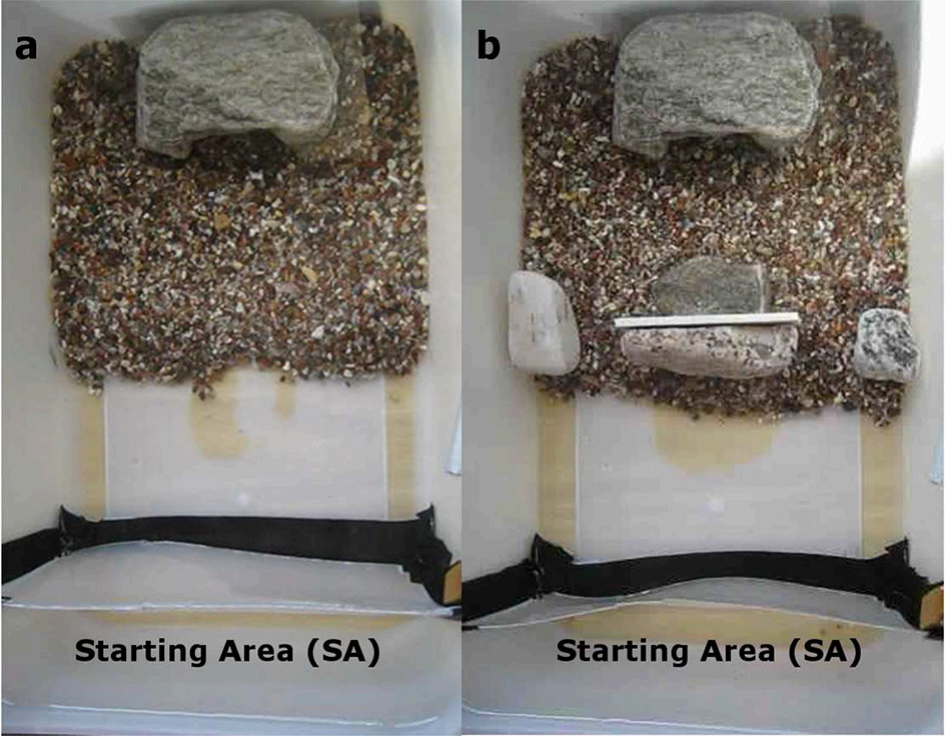
**Experiment 2 - “Rock Fence”: training setup (a)** and test setup **(b)**. The experimental tank is divided into 2 areas: one half has a sandy bottom and a shelter placed centrally, the other half is empty and comprises the “starting area” (SA). This consists of the first portion of the empty area. In the “Rock Fence” test setup there are two narrow passages in the fence.

The same experimental tank was used for training and testing for the two different experiments. This was a rectangular container made of opaque white plastic 26 × 16 × 11 cm (length × width × height). The tank was arranged into two areas along its long axis: in one half of the tank a sandy bottom and a shelter were provided, the other half of the tank was empty and comprised the “starting area.” Two different experimental setups were used for the two experiments (named for consistency 2 and 3). In Experiment 2, the shelter was placed centrally at the end of the sandy area (Figures 2a,b). A transparent plastic separator was used to constrain the animal in the “starting area,” which consisted of the first portion of the empty area about 3 cm long and as wide as the tank itself (Figures 2a,b). During training only the sand and the shelter were present in the tank (Figure 2a). During the test (Experiment 2), a small “rock fence” was placed at the beginning of the sandy area, between the shelter and the “starting area.” This “rock fence” consisted of 3 small rocks, two smaller ones placed laterally at each side of the tank and a wider one placed centrally. Thus, only two narrow passages over the sand were available to reach the shelter behind the rocks (Figures 2a, **4a**). The shelter was always visible to the animal from its starting position both in between and beyond the rocks. The animal could thus reach the shelter by passing over one of the three rocks or around them through one of the two sandy passages in between them. Any vertical route over one of the rocks was always slightly longer (by 2–6 cm, equivalent to 1.5–4 body lengths) than a route through the sand, as the test always started when the animal had settled on the bottom in the starting area.

**Figure 3:**
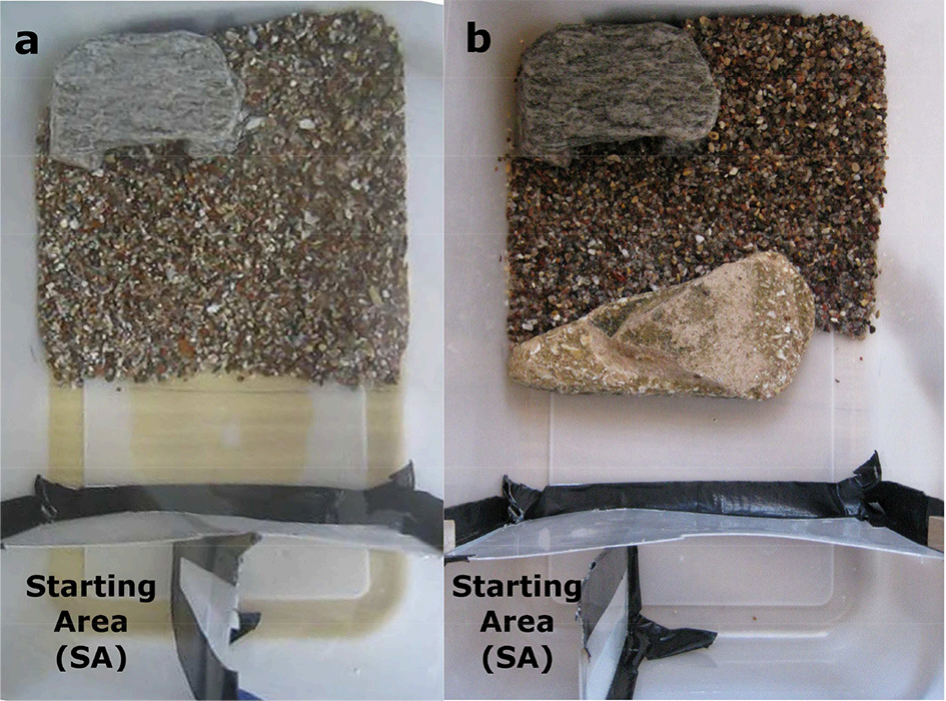
**Experiment 3—“Rock Detour”: training setup (a)** and test setup **(b)**. The experimental tank is divided into 2 areas: one half has a sandy bottom and a shelter placed in the left corner, the other half is empty and comprises the “starting area” (SA). This consists of the left corner of the empty area. In the “Rock Detour” test setup the cuttlefish are released at the left side of the tank and need to go above the rock or all the way around it.

**Figure 4:**
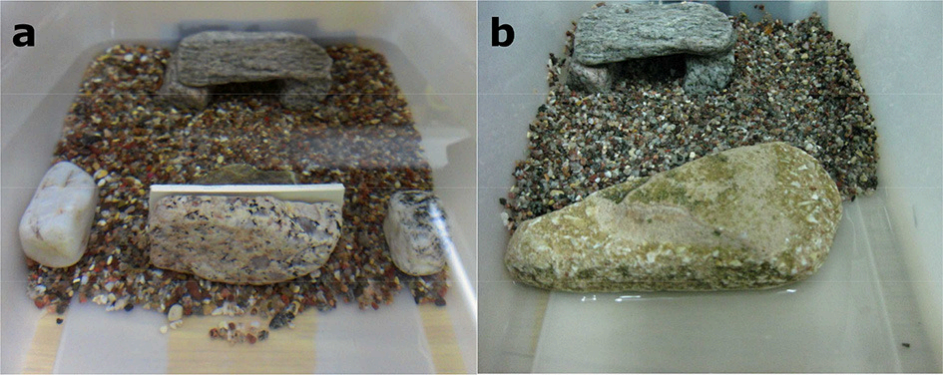
**Front views of the test setups: Experiment 2—“Rock Fence” (a)**, and Experiment 3—“Rock Detour” **(b)**.

In Experiment 3 “rock barrier,” the shelter was placed in the left corner of the sandy area (Figures 3a,b). Two plastic separators were used to delimit the “starting area” at the left corner of the empty area (Figures 3a,b). During training only the sand and the shelter were present in the tank to allow the animals to learn how to reach the latter (Figure 3a). During the test a single rock (13 cm wide) was placed between the start point and the shelter, blocking the left-central area of the tank (Figure 3a). Also in this case, the shelter was visible to the animal if peeking over the top edge of the rock (Figure 4b). The animal could reach the shelter either by swimming straight over the rock (20–25 cm), or by swimming around it (27, 6 cm) through a narrow passage over the sand on the right side of the rock (Figures 3b, 4b). The straight path over the rock was therefore shorter than the horizontal detour around it. All the rocks used as barriers were 2–3 cm tall, which was at least 3 body heights of the animals (Figure S1). Water level was maintained at 10 cm in all experiments, therefore at least 6 cm of water column were available to the animals to swim above the barriers.

#### Training and testing—experiments 2 and 3

The general training and testing procedures were the same for both experiments (Experiment 2, 3). The animals were always tested in the afternoon. Cuttlefish were given 5 training presentations in a row to learn to reach the shelter placed at the opposite side of the experimental tank. In each trial, the animal was placed in the “starting area” (SA) at one side of the tank, in the empty half of the apparatus. Once the animal had settled and after at least 30 s, the transparent wall was raised, and the animal was allowed to move to the shelter. Once the animal had reached the shelter, it was rewarded with 15 min rest in it.

After 5 training runs, a test trial was given. The test trial was performed as the training, but a rock barrier was placed in between the starting point and the shelter. This barrier consisted of a small rock fence (Experiment 2) or a wide rock (Experiment 3). The animal route to the shelter was recorded. The animal was given 15 min rest in the shelter as a reward once it had reached it.

All training and test trials were video-recorded.

#### Experimental setup—night observation experiment

**Figure 5:**
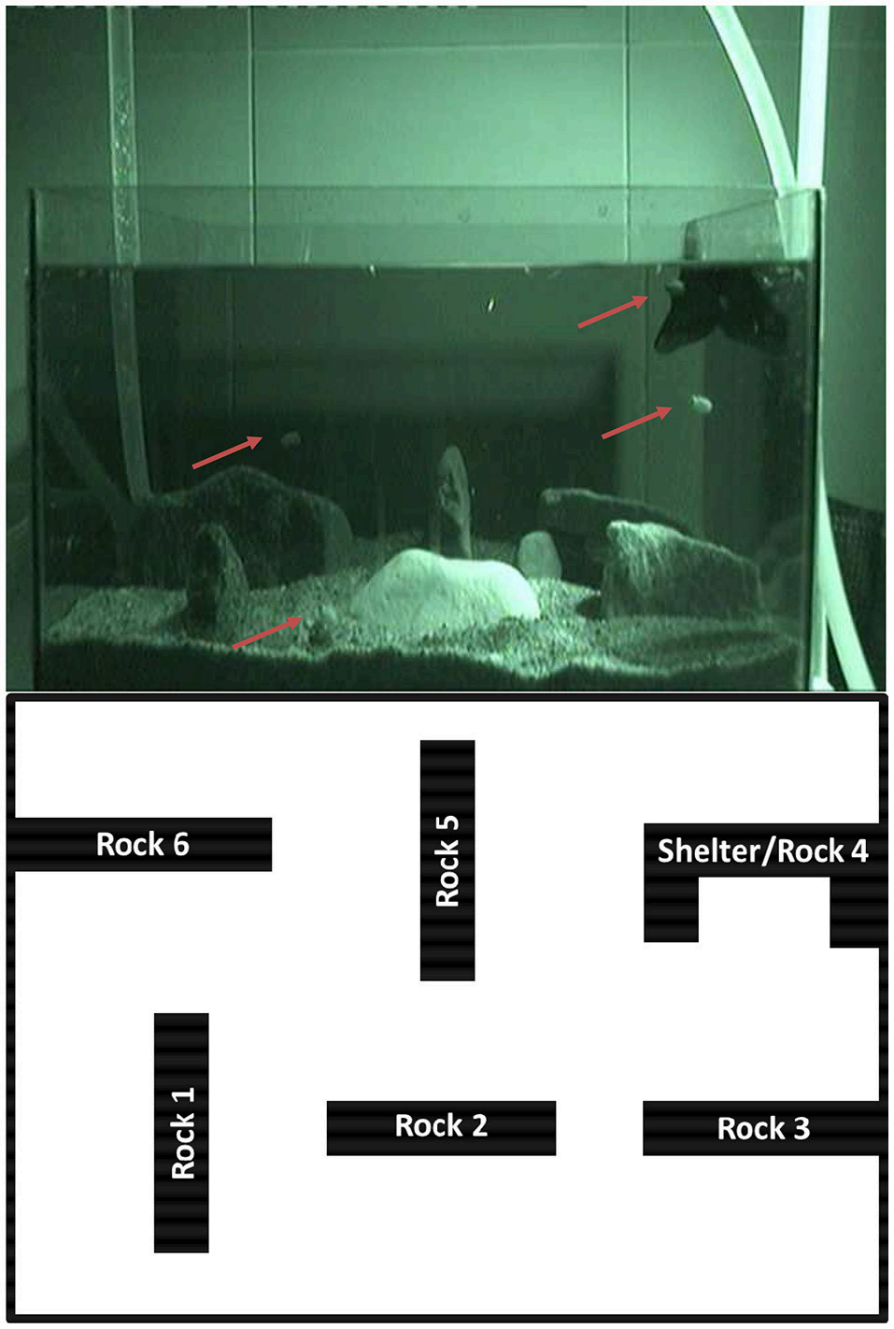
**Night observation experiment**. Video image of four juvenile *Sepia gibba* cuttlefish hunting at night, video recorded with infrared lights. Cuttlefish can be seen using the entire water column during hunting (above). Scheme of the tank used for the night observation providing a top view of the 6 rocks arrangement (below).

A tank (40 × 18 × 25 cm, length × width × height) was filled with sea sand, 5 rocks and a shelter made of rocks. All rocks were tall about 4–12 body heights the cuttlefish (Figure 5). Water level was maintained at 20 cm. The animals were placed in the tank before sunset, and fresh live *Artemia* was added so that the animals could eat at libitum. The behavior of the animals was video-recorded overnight with an infrared camera and light both positioned in front of the tank.

For the analysis, we divided the video-image of the tank in 3 equal depth levels (level 1, level 2, level 3). The top border of level 1 matched the top of the tallest rocks. We recorded the percentage of time each cuttlefish spent in each of the three levels for the first 3 h 30, as nocturnal animals are most active in the first hours after sunset. In addition, we also recorded for each cuttlefish the time spent settled on the bottom, settled on one of the rocks, at the surface and the time in which the animal was not visible because it was behind one of the rocks.

### Statistical analysis

A non–parametric Binomial test with even (0.5) expectancy was used to assess whether the animals' choices during the tests deviated from chance. A *t*-test was used to assess whether there was a difference between the mean time to reach the shelter during the last training trial and the test in Experiment 1 and 2. In Experiment 3, as data were not normal, a Wilcoxon signed-rank test was used to assess whether the mean time to reach the shelter during the last training trial differed significantly from the time needed during the test. As for the night observation experiment, for the values related to the last 2 h of the analyzed video, when animals were clearly distinguishable from each other, a one-way repeated measures Anova was used to compare the percentage time spent at each level. A paired *t*-test was used to compare only the time spent at the first level with the time spent at the second and third levels pooled together. Statistical analyses were performed using R. version 0.98.501 (RStudio, Boston, MA, USA).

## Results

### *S. officinalis* experiments

#### Experiment 1—“rock obstacles”

All 16 young *S. officinalis* cuttlefish moved to the shelter during training and testing. However, while in the first training session it took them on average 9.33 ± 4.5 min (mean ± SD), on the last one they did it in under 5 min (4.67 ± 2.4 min; *t*-test *p* < 0.01). In test runs it took them a little longer but still similar to the last training session (5.25 ± 3.58 min; *t*-test *p* > 0.5). All but one cuttlefish chose the longer yet closer to the ground path (ex. Figure 1b), with a single animal going over the top of a rock and swimming rapidly into the shelter.

### *S. gibba* experiments

#### Experiment 2—“rock fence”

**Figure 6:**
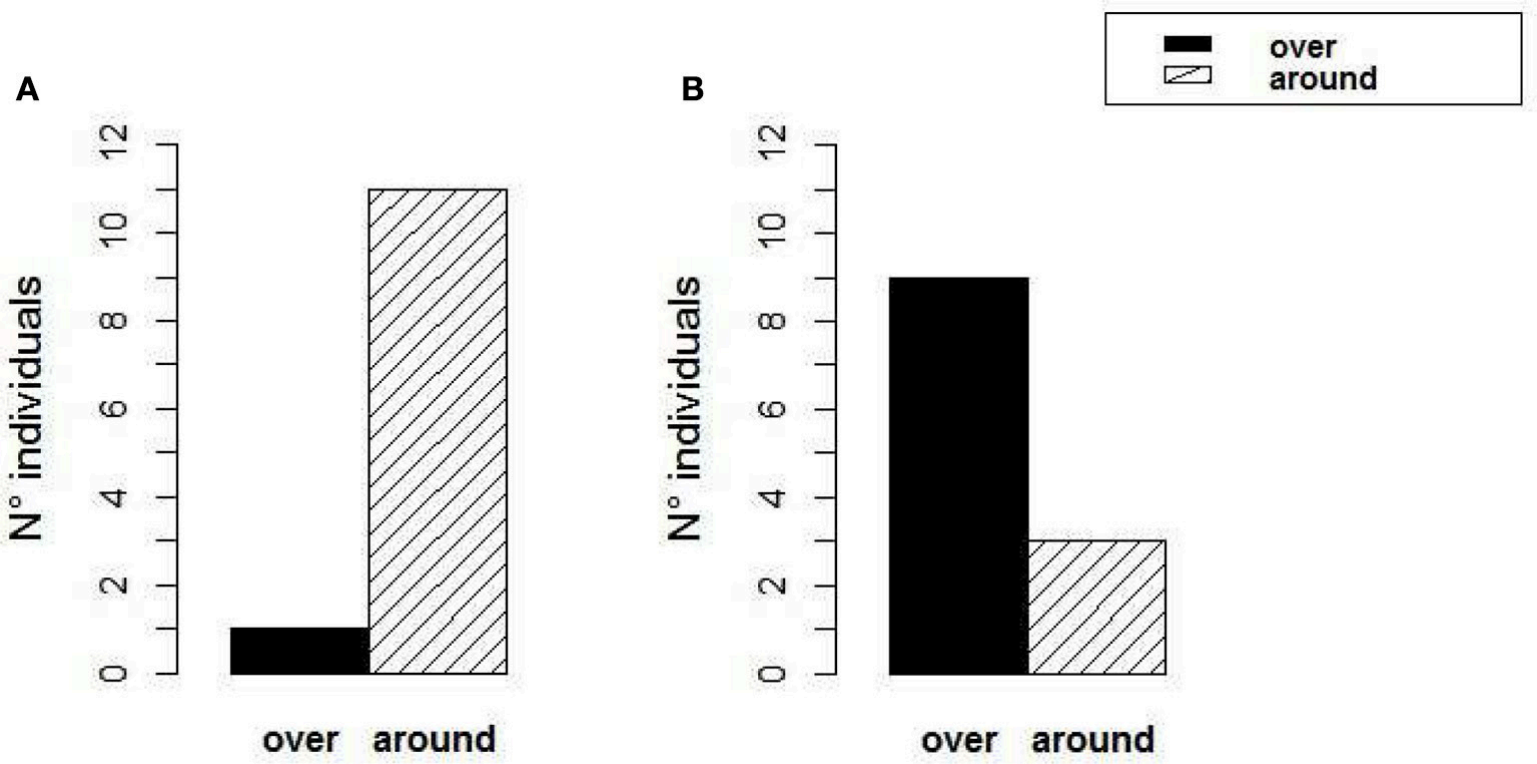
*****Sepia gibba*** choices. (A)** Experiment 2—“Rock Fence”: 11 out of 12 animals chose to reach the shelter through one of the two sandy passages around the rocks, while one animal swam over one of the rocks. **(B)** Experiment 3—“Rock Detour”: 9 out of 12 animals chose to reach the shelter by swimming over the rock, and only 3 animals made a detour around the right edge of the rock and reached the shelter via the sandy passage on the right.

During the test runs, 11 out of 12 animals swam around the rocks, although 3 animals swam at the same height as the top edge of the rocks or immediately below it (Figure 6A) (Binomial test: *p* < 0.001). Only one of the 12 animals swam to the shelter by hovering over the left edge of the rock on the right. One animal, despite choosing to swim over the sandy passage on the right, reached it by swimming at the edge of the rock barrier; therefore it definitely swam higher than the rocks. Most animals selected the sandy passage most close to their starting position, where they had settled before. Only one animal started from the left corner of the starting area and swam to the shelter via the sandy passage on the right instead of using the closest one on the left. Animals took a mean of 10.9 ± 6.7 s (mean ± SD) to reach the shelter in the last training trial, and 9.27 (± 3.4 SD) seconds in the test trial. There was no significant difference between the time needed to reach the shelter in the last training trial and in the test (*t*-test *p* = 0.4). We excluded from the calculation of these mean values the latency to reach the shelter needed by an individual which stopped at the rock for 43 s before moving to the shelter during the test run. This animal took 58 s in total to reach the shelter.

#### Experiment 3—“rock detour”

During the test runs, nine out of 12 animals swam over the rock while 3 animals swam around the rock via the sandy passage on the right side (Figure 6B, Binomial test: *p* = 0.073). Since the animal started always from the left corner of the empty half of the tank, swimming over the left-central part of the rock represented the shortest route to the shelter. Of the 9 animals that swam over the rock, 5 swam over its central part—which was the shortest way, 2 over its left edge and 2 over its right edge. Only 5 out of the 9 animals who swam over the rock went on a straight path directly from their starting position to the shelter over the left-central part of the rock. However, it seems that the remaining 4 animals selected the shortest route possible from the point from which they started heading toward the shelter crossing the rock barrier. Animals took a mean of 10.45 (± 5.4 SD) seconds to reach the shelter in the last training trial, and 26.18 (± 16 SD) seconds in the test trial (Wilcoxon signed-ranks test: *p* < 0.05). We excluded from the calculation of these mean values, one animal which took 6 min and 17 s to reach the shelter in the test, as it settled at the rock barrier for a long time.

#### Night observation experiment

During the first hour and a half, at least two animals were not visible and behind the same rock at the same time; hence for this period we pooled the data of the 4 animals together and calculated a total percentage of time for each parameter. For the remaining 2 h, the 4 animals were always discriminable and time periods were calculated for each of them separately.

Animals spent most of the time in the lowest water level. In the first hour and a half animals spent a total of 83.2% of time in level one (the lowest level), 7.4% of time in the middle level (level two) and 9.2% of time in the highest level (level three) (Table 1). In the following 2 h, animals spent a mean time of 64.8 ± 21.5 % in level 1, 18 ± 10.9 %in level 2 and 17 ± 17.78% in level 3. There was a significant difference among levels (One-way repeated measures ANOVA: F0.05(1),2,3 = 6.62, *p* < 0.05). However, there was no difference between time spent in the first level and time spent in the second and third levels when these last two levels were considered together [paired *t*-test: *t*_(3)_ = 1.3, *p* = 0.26].

**Table 1:** **Percentage of time spent by ***S. gibba*** cuttlefish at each water level (Level 1, 2, 3) and settled on bottom or on rocks, at the water surface or behind rocks during the night observation experiment**.

**Time**	**Level 1**	**Level 2**	**Level 3**	**Settled on bottom**	**At water surface**	**Settled on rocks**	**Behind rocks**
**0:00 -1:30**	83.23	7.48	9.2	7	0.7	4.5	31
**1:30 -3:30**	64.8 ± 21.5	18 ±10.9	17 ±17.78	23 ± 25	4.8 ± 7	15.9 ± 8	18 ± 21

*Data from the first hour and a half were pooled for all 4 cuttlefish*.

During the first hour and a half, animals spent 7% of time settled on the bottom. However, since animals were invisible to us behind one of the rocks for 31% of the time, we could not know whether in this period they were settled on the bottom or moving around near the bottom (always within the first level). During the following 2 h, animals spent a mean of 23 ± 25% of time settled on the bottom, but were invisible to us behind one of the rocks for 18 ± 21% of the time. Animals spent also 4.8 ± 7% of time at the surface and 15.9 ± 8% of time settled on top of one of the rocks, and one animal even spent 6.6% of time settled on the net wrapping the outflow at the top right side of the tank, a couple of body heights from the water surface. Animals mostly moved to hunt both near the bottom and in mid water or close to the water surface (Figure 5); and curiously, if catching a shrimp in mid water they kept eating the prey while maintaining the very same position in the water column (without moving lower or to the bottom).

## Discussion

In this study we investigated the use of three-dimensional space by cuttlefish. In particular, we examined the relative use of vertical vs. horizontal space in a navigational task in which the animal had to negotiate an obstacle in order to reach a shelter. We performed separate experiments which differed not only in the species examined but also in the availability of a direct horizontal (along the ground) path that led to the shelter. This direct horizontal path was present in the second experimental setup (“Rock Fence”), along with slightly longer routes over the rocks; whereas, in the first and third setups (“Rock Obstacle” and “Rock Detour”) only a longer horizontal detour was available, while the shortest path included a vertical displacement over the rock. We also examined the use of different water depths at night by cuttlefish *S. gibba*.

In the second experiment (“Rock Fence”), 11 of 12 animals reached the shelter by swimming through one of the two sandy passages in between the rocks, and ten out of the them took the passage that provided them with the shortest route to the shelter (the closest passage). In the first (“Rock Obstacle”) experiment 15 out of 16 animals chose the slightly longer path while staying near the bottom. However, in the third experiment (“Rock Detour”), when the single narrow passage over the sand was further away from the animals' starting point, the cuttlefish more frequently swam over the rocks than along the longer horizontal detour. Therefore we conclude that, at least during day time, cuttlefish are more likely to move along the bottom, but will take a vertical path if it significantly shortens their way or if the horizontal path may be perceived as blocked. In the night observation experiment, the animals spent most of the time in the lowest water level (level 1), namely within 12 body heights. However, time spent at the first level was not significantly different from time spent above 12 body heights, in both levels 2 and 3. This suggests that at night, *S. gibba* cuttlefish as *S. officinalis* spend a considerable amount of time also far above the bottom mostly hunting and eating in the water column, but also settled camouflaged on tall structures.

The significantly longer time to reach the shelter required in the Detour but not in the Rock Fence test compared with the last training trial might be due to the reduced visibility of the shelter in the Detour test. While in the Rock Fence test cuttlefish could see the shelter from its bottom to its top, in the Detour setup they could only see its top portion behind the rock and might have needed more time to recognize it. In addition, in this case the direct path to the shelter comprised a vertical component thus it was longer than the direct path on the ground of the training run. Alternatively, cuttlefish might have moved slower during the Detour test compared to the training. Unfortunately, we could not assess the animal speed as the cuttlefish movement was recorded only from above and the animal moved both horizontally and vertically. In detour experiments with a vertical component, rats showed a preference for the horizontal-first path over the vertical-first path when both paths were equal in length. However, when the length of the previously preferred horizontal-first route was increased, rats climbing upwards to the goal chose each path equally often (Jovalekic et al., 2011). Other surface-bound species, such as ants and humans, also select routes that include a vertical displacement only when their energetic cost is less than that of an alternative horizontal route (Denny et al., 2001; McNeill Alexander, 2002; Wall et al., 2006; Layton et al., 2009; Holt and Askew, 2012). The energetic cost for vertical and horizontal locomotion in cuttlefish has not yet been thoroughly investigated. However, since cuttlefish and Nautilus have a similar buoyancy system, we can assume that, as in Nautilus (Webber et al., 2000), the cost of vertical movement is similar to that of horizontal swimming (Webber et al., 2000; Aitken and O'Dor, 2006). Hence, since in the Rock Detour the vertical route over the rock was shorter than the horizontal detour, it might have been the less energetically costly. However, in the “Rock obstacles” experiment cuttlefish chose the horizontal route even if this was slightly longer and thus more “expensive” than the vertical. Optimal path choice also depends on factors other than distance and energetic cost, such as predation risk and resource distribution (Makin et al., 2012; Shepard et al., 2013; Sparks et al., 2013). For example, wood ants *Formica rufa* prefer vertical to horizontal detours when these are equal in length (Denny et al., 2001), and this might be associated with the fact that their aphids preys galleries are spread vertically within the canopy (Skinner, 1998). In our case, the cost of using a vertical path should be regarded mainly in terms of safety: exposure to predators by moving up vs. longer exposure by being out of a shelter. The cost of a slightly longer horizontal detour was still lower than the predation cost associated with a vertical route in the Rock obstacles test, but not in the Detour test. Cuttlefish use crypsis as main anti-predatory tactic and as long as they stay camouflaged on the bottom they are hard to detect by predators even when these pass just over them (Hanlon and Messenger, 1988; Staudinger et al., 2013). Direct observations of cuttlefish antipredator behavior in the wild are rare, thus it is hard to assess whether the short vertical distances cuttlefish moved in this study are relevant to predation. However, cuttlefish have demersal and benthic fish threatening them even within the first meter above the seabed (Hanlon and Messenger, 1988; Guerra, 2006), and high predation risk is perceived when a predator swims 6 body heights above the animal (about the range of the vertical movement in this study) (Okamoto et al., 2015). Therefore, even small displacements upwards in the water column can enhance predation risk. In these experiments we used two different cuttlefish species. *Sepia officinalis* inhabits sandy, or rocky substrates and seagrass areas (Guerra, 2006; Guerra et al., 2016), whereas *Sepia gibba* is found in coral reefs, which are among the most complex marine habitats not only along the horizontal but also in the vertical plane (Luckhurst and Luckhurst, 1978; Reid, 2005; Tokeshi and Arakaki, 2012). The availability of preys and shelters in the vertical plane is likely higher in coral reefs than in the habitat of *S. officinalis*. Therefore, we expected *S. gibba* to be more prone to use the vertical space than S. officinalis. However, each species was given its own set of experimental tasks. Under such diverging conditions it is hard to come to clear cut conclusions on habitat-driven behavioral differences or to expand conclusions to an entire genus. In our experiments, both species demonstrated a preference to swimming close to the bottom and while capable of swimming vertically, they did it only adjacent to the surface of the rock. Nonetheless, we showed here that although cuttlefish of both species are basically bottom dwellers, they do use the vertical dimension even by day and move up into the water column when needing to reach a desired location.

Cuttlefish selected paths containing a vertical component more often than the horizontal-only path not only in the Rock Detour when this was much shorter, but also in the “Rock Fence” 3 of the 12 animals moved through the sandy passage by swimming at the same height as the top edge of the surrounding rocks and one of the animals even chose a route over one of the rocks. Also in the “Rock obstacle” experiment one cuttlefish preferred to swim over the rock. Similarly, in the study of Karson et al. (2003), when cuttlefish had to move much higher than in our setup, a few of them still preferred the upper escape hole. Therefore, we believe that these findings (Karson et al., 2003) support our conclusion that, despite being predominantly benthic and preferring to move close to the ground, cuttlefish do also select routes away from the bottom and move in the vertical plane as well. However, as moving vertically away from the bottom is more risky, cuttlefish might need a greater accuracy in the evaluation of positions in this plane. This could explain the preference for vertical information showed in our previous study, where cuttlefish preferentially relied on the correct vertical coordinate rather than on the correct horizontal coordinate of a learned 3D location when these were in conflict (Scatà et al., 2016). The fact that cuttlefish do use the vertical space not only at nightime, but also when needed during the day, suggests that vertical space may be quite important for these animals. High contrast visual cues in the vertical plane seem to be more relevant to a camouflaging cuttlefish than horizontal ones (Mäthger et al., 2006; Ulmer et al., 2013), possibly because masquerading as a nearby object is more effective than blending to the substrate (Buresch et al., 2011). Thus, cuttlefish may as well remember the position of vertical structures in their environment to return to specific locations for effective camouflage or shelter. For example, a single cuttlefish was followed in the field swimming up 3 m to overcome a vertical wall and reach a crevice behind it (Jozet-Alves et al., 2014). Our results are corroborated by a recent preliminary study, when laboratory-reared *Sepia officinalis* cuttlefish were observed using shelters at different heights along the water column (up to 7 body heights higher) and moving to such shelters even during the day (G. Scatà, N. Shashar, and C. Jozet-Alves unpublished results, obtained during a COST Action FA1301 - STSM project). This also suggests that even cuttlefish species living in less complex environments could quickly adapt to vertical structures when available. Cephalopods have highly flexible behavior and they do not seem to need complex habitats for example to express sophisticated camouflage (Shohet et al., 2007; Bush et al., 2017).

*S. officinalis* cuttlefish are mostly active at night, when they move upwards, most likely to forage (Denton and Gilpen-Brown, 1961; Castro and Guerra, 1989; Guerra, 2006; Wearmouth et al., 2013). We observed such behavior also in lab-reared juvenile *Sepia gibba* cuttlefish, which at night moved often upwards to hunt, even all the way to the surface, and settled from time to time on top of the tall rocks (4–12 body heights). Our experiments were conducted during daytime and under constant artificial light conditions and thus may have inhibited the upward movement displayed naturally at night by cuttlefish (Denton and Gilpen-Brown, 1961; Wearmouth et al., 2013). For example, cockroaches that are nocturnal like cuttlefish, tunnel underneath an obstacle in light conditions but climb over it in the dark (Harley et al., 2009). However, at least in captivity, *S. officinalis* spend most of the night close to the water surface with little or no return to the bottom, while during the day remain on the bottom with occasional upwards trips (Wearmouth et al., 2013). In our experiment *S. gibba* cuttlefish did not spend most of the analyzed night time close to the water surface as described for *S. officinalis*. This could be due to different experimental conditions: in our study the animals were provided with *ad libitum* food throughout the night and day, therefore the need to hunt might have been reduced in our animals compared to the *S. officinalis* cuttlefish used in previous studies (Denton and Gilpen-Brown, 1961; Wearmouth et al., 2013); in addition we also used juvenile instead of adult cuttlefish which might as well show a different behavior. However, they did spend similar amount of time close to the bottom as in the upper water levels, and relatively little time settled on the bottom. Therefore, it seems more appropriate to study shelter seeking during daytime when this behavior is more natural.

Nonetheless, cuttlefish seem to change their behavioral patterns between night and day, and vertical movement may be more important at night.

We believe that the relatively low water level (20 times the animals bh), and the space above the rocks (about 12 bh) was enough to allow natural swimming behavior over the rocks. Indeed, some cuttlefish in our study did move even all the way to the surface in the experimental tank before heading to the shelter. Thus, the animals were not inhibited to move upwards by the shallow water. However, it is possible that in deeper waters cuttlefish swim more frequently over vertical obstacles. Further studies are needed to investigate whether this is the case and to explore vertical space use during other behaviors and day/night conditions.

## Ethics statement

The experiments were carried out in accordance with the recommendations of the French and Israeli National Legislation for animal experiments and with the recommendations of the EU directive 2010/63 on the protection of animals used for scientific purposes (Fiorito et al., 2014; Smith et al., 2013). The study was approved by the Committee for experimental with animals, Ben Gurion University, Israel.

## Author contributions

Conceptualization: NS, GS, LD, AD; Methodology: NS, GS, AD; Investigation: NS, GS, SM; Formal analysis: GS and NS; Writing: GS, NS, LD, AD; Funding acquisition: NS, LD, AD; Resources: NS, LD, AD; Supervision: NS and LD

## Fundings

This research did not receive a specific grant from any funding agency in the public, commercial or not-for-profit sectors. NS was an invited scholar at the Univ. of Caen, Normandy, France, during part of the study. GS was supported by a Master's degree scholarship from Ben Gurion University of the Negev, Eilat, Israel.

### Conflict of interest statement

The authors declare that the research was conducted in the absence of any commercial or financial relationships that could be construed as a potential conflict of interest.
