# Supplementary material for: Going Up or Sideways? Perception of Space and Obstacles Negotiating by Cuttlefish
Source: Front Physiol. 2017 Mar 27;8:173. doi: 10.3389/fphys.2017.00173 (PMC5366338; doi:10.3389/fphys.2017.00173)
Supplement: Supplementary file 1 [file DataSheet1.docx]

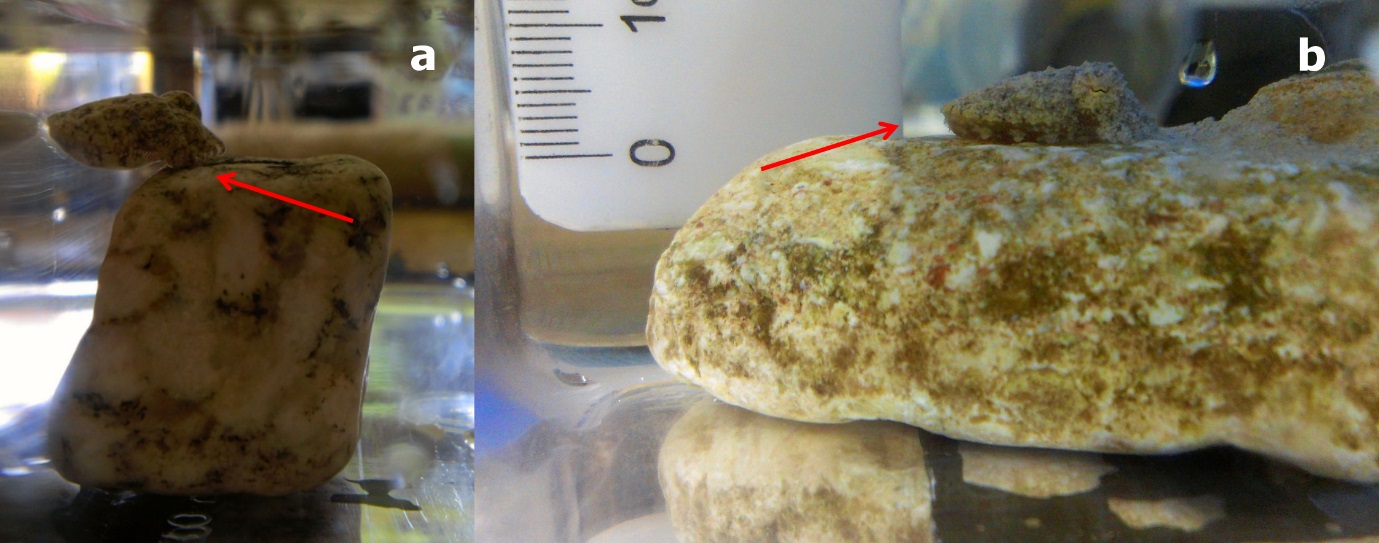


**Figure S1.** A juvenile *Sepia gibba* cuttlefish and barrier rocks used in Experiment 2 (Rock Fence) (a) and Experiment 3 (Rock Detour) (b). The barrier rocks were tall at least 3 body heights of the animal.
